# The clinical, neuropsychological, and brain functional characteristics of the ADHD restrictive inattentive presentation

**DOI:** 10.3389/fpsyt.2023.1099882

**Published:** 2023-03-01

**Authors:** Zhao-Min Wu, Peng Wang, Juan Liu, Lu Liu, Xiao-Lan Cao, Li Sun, Li Yang, Qing-Jiu Cao, Yu-Feng Wang, Bin-Rang Yang

**Affiliations:** ^1^Shenzhen Children's Hospital, Shenzhen, China; ^2^Cardiac Rehabilitation Center, Fuwai Hospital, CAMS and PUMC, Beijing, China; ^3^Peking University Sixth Hospital/Institute of Mental Health, National Clinical Research Center for Mental Disorders (Peking University Sixth Hospital), Beijing, China; ^4^Key Laboratory of Mental Health, Ministry of Health, Peking University, Beijing, China

**Keywords:** ADHD–RI, behavior, cognition, brain imaging, functional connectivity

## Abstract

**Objectives:**

There is an ongoing debate about the restrictive inattentive (RI) presentation of attention deficit hyperactivity disorder (ADHD). The current study aimed to systematically investigate the clinical, neuropsychological, and brain functional characteristics of children with ADHD restrictive inattentive presentation.

**Methods:**

A clinical sample of 789 children with or without ADHD participated in the current study and finished clinical interviews, questionnaires, and neuropsychological tests. Those individuals with a diagnosis of ADHD were further divided into three subgroups according to the presentation of inattentive and/or hyperactive/impulsive symptoms, the ADHD-RI, the ADHD-I (inattentive), and the ADHD-C (combined) groups. Between-group comparisons were carried out on each clinical and neuropsychological measure using ANCOVA, with age and sex as covariates. Bonferroni corrections were applied to correct for multiple comparisons. Two hundred twenty-seven of the subjects also went through resting-state functional magnetic resonance imaging scans. Five ADHD-related brain functional networks, including the default mode network (DMN), the dorsal attention network (DAN), the ventral attention network, the executive control network, and the salience network, were built using predefined regions of interest (ROIs). Voxel-based group-wise comparisons were performed.

**Results:**

Compared with healthy controls, all ADHD groups presented more clinical problems and weaker cognitive function. Among the ADHD groups, the ADHD-C group had the most clinical problems, especially delinquent and aggressive behaviors. Regarding cognitive function, the ADHD-RI group displayed the most impaired sustained attention, and the ADHD-C group had the worst response inhibition function. In terms of brain functional connectivity (FC), reduced FC in the DMN was identified in the ADHD-C and the ADHD-I groups but not the ADHD-RI group, compared to the healthy controls. Subjects with ADHD-I also presented decreased FC in the DAN in contrast to the control group. The ADHD-RI displayed marginally significantly lower FC in the salience network compared to the ADHD-I and the control groups.

**Conclusion:**

The ADHD-RI group is distinguishable from the ADHD-I and the ADHD-C groups. It is characterized by fewer externalizing behaviors, worse sustained attention, and better response inhibition function. The absence of abnormally high hyperactive/impulsive symptoms in ADHD-RI might be related to less impaired brain function in DMN, but potentially more impairment in the salience network.

## Introduction

Attention-deficit/hyperactivity disorder (ADHD) has a relatively high prevalence among children and adolescents, making it one of the most common neurodevelopmental disorders. Worldwide estimations of its prevalence in children ranged from 3 to 7% (1). The core symptoms of ADHD were divided into two domains in the Diagnostic and Statistical Manual of Mental Disorders, fifth edition (DSM-5) (2), the inattention domain and the hyperactivity/impulsivity domain. Each domain includes nine symptoms, and the presence of six or more symptoms in either domain will lead to the diagnosis of ADHD. According to the number of symptoms in each domain, ADHD patients can be clarified into three different presentations based on DSM-5 (2), the predominantly inattentive presentation (ADHD-PI), the predominantly hyperactive/impulsive presentation (ADHD-HI), and the combined presentation (ADHD-C).

The ADHD symptoms do not have developmental stability. In preschool years, the hyperactive/impulsive symptoms dominated, while the inattention symptoms became more obvious by the age of 6–7 years (3). A previous study investigated the causal relationship between these two ADHD symptom domains. What they found in their data suggested that inattention is a driving factor for hyperactivity/impulsivity (4). There is also evidence that shared, and unique neuropsychological characteristics, brain structure, brain function, and genetic risks underlie the two symptom domains (5–7). A longitudinal study found no significant correlation between the degree of ADHD symptom reduction and the magnitude of neuropsychological function improvement (8). In that study, the severity of the hyperactive/impulsive symptom domain declined more than the inattentive symptom domain (52.79 vs. 22.03%). From all the evidence from prior studies, we can infer that the two ADHD symptom domains are distinct albeit intertwined with each other, which leads to different clinical, and neuropsychological characteristics, as well as distinct neural correlates of different ADHD presentations.

Nevertheless, previous studies investigating the difference between the ADHD-PI and the ADHD-C subtypes generated inconsistent results. Some studies revealed that those patients with ADHD-C had more severe clinical impairments, albeit less cognitive dysfunction, than the ADHD-PI patients in some cognitive domains (9, 10), e.g., information processing speed and sustained attention. It has been shown that ADHD presentations did not fall into a severity continuum. In fact, the ADHD-PI group is highly heterogenous since some of those patients with ADHD-PI present subthreshold hyperactive/impulsive symptoms. Therefore, some scientists suggested a new presentation of ADHD, the restricted inattentive presentation (ADHD-RI), which requires six or more inattentive symptoms and fewer than three hyperactive/impulsive symptoms (11). Individuals with ADHD-RI are “purely” inattentive, with normal hyperactivity/impulsivity levels. The ADHD-RI presentation allows us to investigate the “pure” effects of the inattentive symptoms and the effects of hyperactive/impulsive symptoms on the inattentive domain when comparing to the ADHD-I (those with six or more inattentive symptoms and three to five hyperactive/impulsive symptoms) and the ADHD-C presentations.

A few studies have, in fact, investigated the clinical and neuropsychological characteristics, as well as the neural correlates of this new ADHD presentation. A clinical study of 87 adolescents with ADHD showed that the ADHD-RI patients scored lower than those with ADHD-I in two factors from the conners’ parent rating scale (CPRS), that is, hyperactivity and learning problems (12). A study on a group of (*n* = 145) adolescents with or without ADHD demonstrated more impaired early-stage attention control and less impaired late-stage response inhibition in individuals with ADHD-RI than the ADHD-C subjects (13). Another study (*n* = 301) also detected lower psychomotor speeds, and longer reaction time during neuropsychological tests in individuals with ADHD-RI, compared to healthy controls, the ADHD-C, and the ADHD-I type (14). Upon using the task-based fMRI technique (*n* = 96), the ADHD-RI group was found to activate more attention-related posterior brain regions (especially the temporo-occipital areas) compared to healthy controls and the ADHD inattentive type (14).

Although we have noticed the value of investigating the ADHD-RI presentation, there are few studies doing so. In addition, the sample sizes of existing studies are relatively small. Some of the studies utilized non-clinical samples, which might restrain the generalization of the findings. The current study, therefore, aimed to systematically explore the clinical, neuropsychological, and brain functional characteristics in a relatively large sample size clinical cohort of children with and without ADHD (all medication naïve, total *n* = 789, with brain imaging *n* = 297). We hypothesized that patients with ADHD-RI displayed fewer clinical problems, more impaired sustained attention, less impaired inhibition function, and a different functional connectivity pattern compared to patients with ADHD-I, patients with ADHD-C, and the healthy controls.

## Methods

### Participants

A total of 681 children with ADHD (aged 6–15 years) were recruited in the child health care and mental health center of Shenzhen children’s hospital. Another 108 typically developing children were recruited from local elementary schools. All of the participants and their parents were interviewed before enrollment. The diagnosis of ADHD and/or any other psychiatric disorder was confirmed or excluded through a clinical interview and a semi-structured interview based on the Schedule for Affective Disorders and Schizophrenia for School-Age Children -present and lifetime version (K-SADS-PL) (15) according to the Diagnostic and Statistical Manual of Mental Disorders Fourth Edition (DSM-IV) (16). The inclusion criteria for the ADHD group include: (1) aged 6–15 years; (2) educated in public/ordinary private schools; (3) a diagnosis of ADHD. As for the control group, the age and education requirements were the same as for the ADHD group. The exclusion criteria for both the ADHD and the control groups include (1) a history of head injury with loss of consciousness; (2) any kind of severe physical disease or neurological abnormalities; (3) any kind of drug or substance misuse; (4) a full-scale IQ measured by Wechsler Intelligence Scale for Chinese Children-IV (WISC-IV) below 70; (5) Long-term use of any prescribed medications for ADHD or other medical conditions. For those who participated in the MRI scans, they were all right-hand dominant, and any visible abnormalities (e.g., arachnoid cyst) on the MRI images or a past or current history of claustrophobia would lead to exclusion. Flow charts of subject inclusion and exclusion can be found in the supplementary material. The ADHD group was divided into three subgroups according to their symptom presentations revealed in the K-SADS-PL interviews. The three ADHD subgroups were (1) ADHD-RI: ADHD restrictive inattentive presentation, with six or more inattentive symptoms and fewer than three hyperactive/impulsive symptoms; (2) ADHD-I: ADHD inattentive presentation, with six or more inattentive symptoms and three to five hyperactive/impulsive symptoms; (3) ADHD-C: ADHD combined presentation, with six or more inattentive symptoms and six or more hyperactive/impulsive symptoms. Besides, the summing severity scores of each symptom from the K-SADS-PL interview were used as indicators of symptom severity in subsequent statistical analysis.

This work was approved by the Ethics committee of Shenzhen Children’s Hospital. Informed consent was obtained from parents and children.

### Clinical and cognitive assessments

Besides the clinical and semi-structured interviews, a battery of clinical and cognitive assessments was carried out on both ADHD patients and healthy controls. The Child Behavior Checklist (CBCL; (17)) was filled by the parent who knows the child best. To capture the executive function of each individual, the Wechsler Intelligence Scale for Chinese Children-IV (WISC-IV) and several tests from the Cambridge Neuropsychological Test Automated Battery (CANTAB) were performed, including Spatial Working Memory (SWM), Stop Signal Task (SST), Rapid Visual Information Processing (RVP) and Reaction Time (RTI) were conducted by well-trained psychologists or nurses.

The Child Behavior Checklist (CBCL) (17): it contains 113 items to assess the daily behavior of each individual. All items were summarized into eight factors, including withdrawn, somatic complaints, anxiety/depression, social problems, thought problems, attention problems, delinquent behaviors, and aggressive behaviors. It has been widely used in clinical settings and scientific research (10). More information on this scale can be found in its manual. The original scale has been translated into Chinese, and the reliability and validity have been tested (18). Since the standardized t-scores were not available in mainland China, the original summary score of each factor was used in the current study.

The Wechsler Intelligence Scale for Chinese Children-IV (WISC-IV): it has been widely used to evaluate the intelligence of children aged 6–16 years (19). All administrators of WISC-IV have to be systematically trained. Results of WISC-IV include a full-scale intelligence quotient (FIQ), and 4 index scores, e.g., the Verbal Comprehension Index (VCI), the Perceptual Reasoning Index (PRI), the Working Memory Index (WMI), and the Processing Speed Index (PSI).

The CANTAB: the CANTAB has been widely used in scientific research about all kinds of psychiatric disorders, including ADHD (20). In the current study, we utilized cloud-based products, which means all instructions were administered automatically. All participants were required to finish several tasks. More detailed information about these CANTAB tasks and their major measures can be found on their website.^1^ Brief information about these chosen tasks is also listed below.

1. Spatial Working Memory (SWM) is a test of retention and manipulation of visuospatial information. There are a number of boxes shown on the screen, and a “token” will be hidden in one of the boxes at a time. The “token” will not be hidden in the same box twice, which means the participants have to remember the positions of the boxes where the token has been taken. The major outcome measures include SWMS (strategy, it reflects the possibility of the participant using a certain searching strategy), and SWMBE (between errors, the number of times an individual incorrectly revisits an emptied box).
2. Stop Signal Task (SST) is a test of response inhibition. This test asked the participants to inhibit their responses to an arrow stimulus once they hear the stop signal (a beep). The major outcome measures include the SSTSSRT (stop signal reaction time), the SSTDEG (direction errors: go trials), and the SSTDES (direction errors: stop trials).
3. Rapid Visual Information Processing (RVP) is a test of sustained attention and information processing speed. Generally, the participants are asked to hit on the screen when they spot the targeted sequence of digits in a box, where digits appear one by one in pseudo-random order. The major outcome measures include the RVPA (A prime; This measure reflects the sensitivity to the target regardless of response tendency. Ranged 0.00 ~ 1.00, the higher, the better.), RVPML (the mean response latency), the RVPPFA (the probability of false alarm), and the RVPPH [probability of (correct) hit].
4. Reaction Time (RTI) measures sustained attention and motor and mental response speeds. The participants are asked to hold a button at the bottom of the screen. There are one (the simple mode) or five circles (the five-choice mode) presented above. A yellow dot will appear in one of the circles from time to time. The participants are required to release the button and select the circle with a yellow dot inside as soon as possible. The major outcome measures include the RTISMDRT (reaction time of the simple mode), the RTIFMDRT (reaction time of the five-choice mode), the RTISRTSD (the standard deviation (SD) of reaction time of the simple mode), and the RTIFRTSD (the SD of reaction time of the five-choice mode).

### Imaging protocols

A total of 297 individuals participated in the resting state functional magnetic resonance imaging scans (RS-fMRI), including 87 with ADHD-I, 71 with ADHD-C, 46 with ADHD-RI, and 93 healthy controls. The MRI images were acquired using the 3 T Siemens Skyra scanner with a standard 12-channel head coil located in Shenzhen Children’s Hospital. Single-shot echo-planar imaging (EPI) sequences were applied: TR = 2000 ms, TE = 30 ms, flip angle = 90°, thickness/skip = 3.5/0.7 mm, matrix = 64 × 64, field of view (FOV) = 200 × 200 mm, 33 axial slices, 240 volumes, and 3 mm × 3 mm in-plane resolution.

### Preprocessing of the RS-fMRI images

All the RS-fMRI images were preprocessed in FSL^2^ and Python.^3^ Details about the preprocessing of RS-fMRI images can also be found in our previous publication (21). The following steps were applied: (1) removing the first 10 time points; (2) realigning to the middle volume (head motion correction); (3) performing the grand mean scaling; (4) spatial smoothing at a Gaussian kernel of 6 mm full-width at half-maximum; (5) applying the independent components analysis-based automatic removal of motion artifacts (ICA-AROMA) (22), and removing the head motion components; (6) performing nuisance regression to remove the cerebrospinal fluid and white matter signals; (7) high pass filtering (0.01 Hz); and (8) registering the images to a standard space. A total of four participants (all ADHD subjects) were excluded due to excessive head motion (head motion >3 mm of translation or >3 degrees of rotation in any direction). A summary score of head motion across time, the root mean squared of the relative displacement time series (RMS) from mcflirt (function of FSL) was derived as an indicator of head motion and used as covariates in subsequent between-group analyses.

### Construction of brain networks and between-group comparisons

All brain networks were built using predefined regions of interest (ROI). (1) the default mode network (DMN): this network was built using the posterior cingulate cortex (PCC) as ROI, which was created from the Harvard-Oxford atlas;^4^ (2) the other brain networks were made from predefined 6 mm spherical seeds centered in predefined coordinates: the executive control network: the bilateral dorsal prefrontal cortex (DLPFC; ±44, 36, 20) (23); the salience network: dorsal anterior cingulate cortex (6, 45, 9) and the bilateral anterior frontoinsular cortex (−45, 35, 9 and 45, 3, 15) (24); the dorsal attention network: bilateral intraparietal sulcus (±32, −56, 54) and frontal eye field (±28, −8, 52) (25); ventral attention network: bilateral ventral frontal cortex (±42, 20, 6) and bilateral temporoparietal junction (±60, −48, 22) (25). The six brain networks of interest were shown in the Supplementary Figure S3. The individual functional networks were obtained, and group-wise comparisons were performed using the dual-regression and Randomise frameworks in FSL (26). The threshold-free cluster enhancement (TFCE) was used for cluster-wise statistical corrections, generating *p*-values corrected for whole-skeleton family-wise error (FWE). Only those significant clusters larger than 10 voxels were reported. Although we have mainly adopted a categorical concept of ADHD and examined the between-group differences, alternative dimensional models (with symptom severity as independent variables) and hybrid models (with symptom severity and the interaction between diagnosis and symptom severity as independent variables) were also tested. In addition, sensitivity analyses were performed with age^2 or head motion parameter as an additional covariate. The potential interaction between diagnosis and age was also evaluated. In addition, the brain-behavior and brain-cognition relationships were also investigated. More details about these alternative models can be found in the supplementary material.

### Statistical analysis

All of the statistical analyses were performed in R. Group-wise comparisons of the behavioral and cognitive data were finished by performing ANCOVA, with age and sex as covariates. Bonferroni’s correction was applied by dividing 0.05 by the number of measures from the same scale or tasks. Therefore, the significance level was set to 0.05/8 = 0.00625 for all CBCL factors, 0.05/4 = 0.0125 for measures from the WISC-IV, the RVP task, and the RTI task, 0.05/3 = 0.0167 for the measures from the SST task, and 0.05/2 = 0.025 for the measures from the SWM task.

## Results

### Demographic characteristics

In general, the ADHD-RI group was older than the ADHD-C group, and the control group was older than both the ADHD-I and the ADHD-C groups (all *p* < 0.05). There are more males in all of the ADHD groups compared to the healthy control (HC; *p* < 0.0001). In addition, there are also more males in the ADHD-C group in contrast to the ADHD-I and the ADHD-RI groups (both *p* < 0.001). As expected, the ADHD groups had more inattentive and hyperactive/impulsive (H/I) symptoms than the control group (all *p* < 0.05). Among all the ADHD groups, the ADHD-C group had the highest score in both symptom domains (all *p* < 0.05). Note that, in terms of inattention symptoms, there was no significant difference between the ADHD-RI and the ADHD-I groups (*p* = 0.262). As for the WISC-IV indicators, all of the ADHD groups have lower FIQ, VCI, PRI, WMI, and PSI scores (all *p* < 0.0125). There is no significant group-wise difference among the three ADHD groups in any of the indicators from WISC-IV (all *p* > 0.05). More details can be found in Table 1.

**Table 1:** Demographic information of the ADHD groups and the control group.

	ADHD-RI	ADHD-I	ADHD-C	HC	*p*	Group-wise comparisons
Sample size	128	175	378	108	N.A.	N.A.
No. of males (percentage of males)	102 (79.69%)	141 (80.57%)	344 (91.01%)	61 (56.48%)	<0.0001	HC < ADHD-RI/ADHD-I < ADHD-C^#^
Age	9.16 ± 2.00	8.83 ± 1.76	7.91 ± 1.50	9.43 ± 1.35	<0.0001	HC > ADHD-I/ADHD-C ADHD-RI > ADHD-C
Inattentive symptoms	24.02 ± 1.58	24.34 ± 1.72	24.95 ± 1.68	16.42 ± 2.35	<0.0001	ADHD-RI/ADHD-I/ADHD-C > HC ADHD-C > ADHD-I/ADHD-RI
Hyperactive/impulsive symptoms	13.86 ± 2.16	18.98 ± 1.91	24.29 ± 1.59	11.91 ± 1.14	<0.0001	ADHD-RI < ADHD-I < ADHD-C < HC
FIQ	94.27 ± 10.37	94.02 ± 9.72	94.79 ± 11.19	104.50 ± 9.18	<0.0001	ADHD-RI/ADHD-I/ADHD-C < HC
VCI	96.08 ± 11.95	96.39 ± 10.33	96.93 ± 11.92	102.31 ± 10.12	<0.0001	ADHD-RI/ADHD-I/ADHD-C < HC
PRI	101.52 ± 11.19	100.06 ± 11.29	101.40 ± 12.61	106.47 ± 10.58	0.00028	ADHD-RI/ADHD-I/ADHD-C < HC
WMI	88.84 ± 9.84	89.16 ± 10.06	88.79 ± 10.37	97.48 ± 10.11	<0.0001	ADHD-RI/ADHD-I/ADHD-C < HC
PSI	93.71 ± 11.22	93.88 ± 12.07	94.26 ± 11.43	106.95 ± 11.23	<0.0001	ADHD-RI/ADHD-I/ADHD-C < HC

ADHD-RI, ADHD restrictive inattentive presentation, with six or more inattentive symptoms and fewer than three hyperactive/impulsive symptoms; ADHD-I, ADHD inattentive presentation, with six or more inattentive symptoms and three to five hyperactive/impulsive symptoms; ADHD-C, ADHD combined presentation, with six or more inattentive symptoms and six or more hyperactive/impulsive symptoms; FIQ, full-scale intelligence quotient; VCI, verbal Comprehension Index; PRI, perceptual reasoning index; WMI, working memory index; PSI, processing speed index; HC, healthy controls. The “<” and “>” indicated significant differences and the directions; N.A., not applicable.

# The gender differences between groups were tested using pair-wise and whole group chi square tests, and significant level was corrected for multiple comparisons using Bonferroni correction method.

### Clinical and neuropsychological features

In all of the eight indicators from CBCL except for the somatic complaint and the thought problems factor, all three ADHD groups scored higher than the control group (all *p* < 0.00625). The ADHD-C group scored higher than the control group in the thought problems factor (*p* < 0.001). None of the ADHD groups had a higher or lower score in the somatic complaint factor compared to the control group (all *p* > 0.0625). Among the three ADHD groups, the ADHD-C group had a higher score than the ADHD-RI group in the attention problems factor, the delinquent behaviors factor, and the aggressive behaviors factor (both *p* < 0.00625). The ADHD-C group also scored higher than the ADHD-I group in the aggressive behaviors factor (*p* < 0.001). None of the other group-wise comparisons of the CBCL factors among the three ADHD groups yielded significant results (all *p* > 0.00625). More details can be found in Table 2.

**Table 2:** Clinical profile of the ADHD groups and the control group.

CBCL factors	ADHD-RI	ADHD-I	ADHD-C	HC	*p*	Group-wise comparisons
Sample size	110	133	310	55	N.A.	N.A.
Withdrawn	3.27 ± 2.91	3.26 ± 2.56	2.84 ± 2.40	1.58 ± 1.99	0.00023	ADHD-RI/ADHD-I/ADHD-C > HC
Somatic complaints	1.58 ± 2.11	1.40 ± 1.76	1.43 ± 1.67	0.73 ± 1.48	0.035	N.A.
Anxiety/Depression	3.76 ± 4.05	4.08 ± 3.97	4.28 ± 3.85	1.55 ± 2.54	<0.0001	ADHD-RI/ADHD-I/ADHD-C > HC
Social problems	3.55 ± 2.79	3.99 ± 2.91	4.45 ± 2.62	1.42 ± 1.64	<0.0001	ADHD-RI/ADHD-I/ADHD-C > HC
Thought problems	1.03 ± 1.62	1.09 ± 1.29	1.29 ± 1.58	0.39 ± 1.10	0.00025	ADHD-C > HC
Attention problems	7.57 ± 3.46	8.36 ± 3.10	8.82 ± 3.32	1.81 ± 2.18	<0.0001	ADHD-RI/ADHD-I/ADHD-C > HC ADHD-C > ADHD-RI
Delinquent behaviors	3.27 ± 2.91	3.66 ± 2.48	4.34 ± 2.97	1.41 ± 2.09	<0.0001	ADHD-RI/ADHD-I/ADHD-C > HC ADHD-C > ADHD-RI
Aggressive behaviors	8.17 ± 6.21	10.50 ± 5.75	13.81 ± 6.69	3.64 ± 3.81	<0.0001	ADHD-C > ADHD-RI/ADHD-I > HC

ADHD-RI, ADHD restrictive inattentive presentation, with six or more inattentive symptoms and fewer than three hyperactive/impulsive symptoms; ADHD-I, ADHD inattentive presentation, with six or more inattentive symptoms and three to five hyperactive/impulsive symptoms; ADHD-C, ADHD combined presentation, with six or more inattentive symptoms and six or more hyperactive/impulsive symptoms; HC, healthy controls. The “<” and “>” indicated significant differences and the directions; N.A., not applicable.

In the RVP task, compared to the control group, the ADHD-C group had a smaller RVPA score (*p* = 0.00354), and the ADHD-RI and the ADHD-I group had a higher RVPML (*p* = 0.0072 and 0.013). In the SST task, compared to the control group, the ADHD-I and ADHD-C groups had higher SSTDEGs (both *p* < 0.001) and SSTDESs (*p* = 0.012 and 0.0077 respectively). In the RTI task, compared to the control group, the ADHD-RI group scored higher in RTISMDRT (*p* < 0.001), and RTIFMDRT (*p* < 0.0001). The ADHD-C and ADHD-I group also had higher RTIFMDRT scores than the control group (both *p* < 0.001). The ADHD-C group had a marginally significantly smaller RTISMDRT than the ADHD-RI group (*p* = 0.0176). No other significant group-wise differences were detected. More details can be found in Table 3.

**Table 3:** Neuropsychological characteristics of the ADHD groups and the control group.

CANTAB measures	ADHD-RI	ADHD-I	ADHD-C	HC	*p*	Group-wise comparisons
Sample Size	102	135	290	100	N.A.	N.A.
RVPA	0.78 ± 0.074	0.76 ± 0.088	0.73 ± 0.092	0.79 ± 0.088	0.0028	ADHD-C < HC
RVPML	649.10 ± 157.57	639.01 ± 179.87	637.41 ± 169.21	582.51 ± 107.16	0.0056	ADHD-RI/ADHD-I > HC
RVPPFA	0.23 ± 0.28	0.24 ± 0.26	0.27 ± 0.25	0.17 ± 0.22	0.58	N.A.
RVPPH	0.53 ± 0.25	0.53 ± 0.26	0.51 ± 0.24	0.53 ± 0.20	0.71	N.A.
SWMS	8.67 ± 1.99	8.91 ± 1.57	8.49 ± 2.06	8.33 ± 2.09	0.064	N.A.
SWMBE	19.60 ± 8.57	18.84 ± 8.13	20.78 ± 8.47	1.65 ± 8.65	0.12	N.A.
SSTSSRT	360.53 ± 78.03	381.54 ± 73.67	380.06 ± 104.88	339.45 ± 83.09	0.066	N.A.
SSTDEG	26.27 ± 25.10	30.72 ± 31.72	33.74 ± 28.99	14.09 ± 16.19	0.00014	ADHD-C/ADHD-I > HC
SSTDES	49.45 ± 8.53	50.78 ± 8.48	50.71 ± 8.30	46.86 ± 8.01	0.0071	ADHD-C/ADHD-I > HC
RTISMDRT	465.53 ± 204.00	437.26 ± 104.77	454.93 ± 79.04	380.87 ± 60.36	0.00029	ADHD-RI > HC
RTIFMDRT	502.58 ± 120.96	489.38 ± 80.68	520.01 ± 80.97	428.70 ± 60.93	<0.0001	ADHD-RI/ADHD-I/ADHD-C > HC
RTISRTSD	130.04 ± 180.91	122.01 ± 138.64	133.95 ± 118.23	71.40 ± 37.78	0.12	N.A.
RTIFRTSD	117.53 ± 101.99	106.78 ± 51.20	130.44 ± 93.27	85.10 ± 49.35	0.080	N.A.

CANTAB, the Cambridge Neuropsychological Test Automated Battery; RVPA, Rapid Visual Information Processing (RVP), A prime; RVPML, Rapid Visual Information Processing (RVP), mean response latency; RVPPFA, Rapid Visual Information Processing (RVP), probability of false alarm; RVPPH, Rapid Visual Information Processing (RVP), probability of hit; SWMS, Spatial Working Memory (SWM), strategy; SWMBE, Spatial Working Memory (SWM), between errors; SSTSSRT, Stop Signal Task (SST), stop signal reaction time; SSTDEG, Stop Signal Task (SST), direction errors: go trials; SSTDEG, Stop Signal Task (SST), direction errors: stop trials; RTISMDRT, Reaction Time (RTI), reaction time of the simple mode; RTIFMDRT, Reaction Time (RTI), reaction time of the five-choice mode; RTISRTSD, Reaction Time (RTI), the standard deviation of reaction time of the simple mode; RTIFRTSD, Reaction Time (RTI), the standard deviation of reaction time of the five-choice mode; ADHD-RI, ADHD restrictive inattentive presentation, with six or more inattentive symptoms and fewer than three hyperactive/impulsive symptoms; ADHD-I, ADHD inattentive presentation, with six or more inattentive symptoms and three to five hyperactive/impulsive symptoms; ADHD-C, ADHD combined presentation, with six or more inattentive symptoms and six or more hyperactive/impulsive symptoms; HC, healthy controls. The “<” and “>” indicated significant differences and the directions; N.A., not applicable.

### Functional connectivity

**Figure 1:**
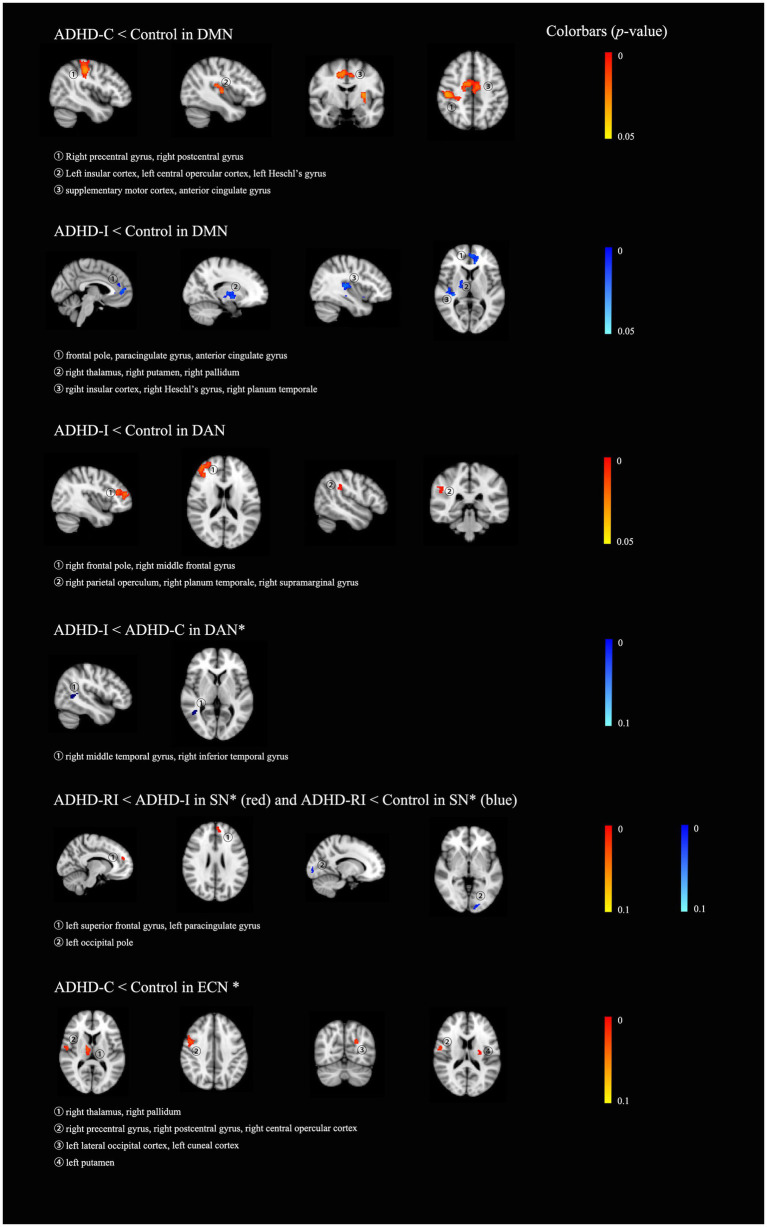
The results of group-wise comparisons among the four groups, the ADHD restrictive inattentive presentation group (ADHD-RI), the ADHD inattentive presentation group (ADHD-I), the ADHD combined presentation (ADHD-C) and the healthy control group (HC). All results were displayed on an MNI_T1_2mm_brain. * indicated that those are clusters marginally significant, that is, with a *p*-value between 0.05 to 1 (0.05 < *p* < 0.1). DMN, default mode network; DAN, dorsal attention network; VAN, ventral attention network; ECN, executive control network; SN, salience network.

In terms of functional connectivity within the DMN, compared with the control group, the ADHD-I group showed decreased functional connectivity mainly in the left frontal lobe, the right insular, the right thalamus, and bilateral putamen, and the ADHD-C showed decreased functional connectivity mainly in the right postcentral gyrus, the right precentral gyrus, the posterior cingulate gyrus, and the left insular. As for the dorsal attention network, the ADHD-I group showed decreased functional connectivity, mainly in the right frontal lobe, compared to the control group. The ADHD-I group also displayed marginally significantly (0.05 < *p* < 0.1) lower functional connectivity in the right temporal lobe compared to the ADHD-C group within the dorsal attention network. Within the salience network, the ADHD-RI group showed marginally significantly (0.05 < *p* < 0.1) decreased functional connectivity in the left occipital pole compared to the control group, as well as in the left superior frontal gyrus, and the left paracingulate gyrus compared to the ADHD-I group. As for the executive control network, the ADHD-C group showed marginally significantly (0.05 < *p* < 0.1) decreased functional connectivity than the control group, mainly in the right thalamus, the right pallidum, the right accumbens, the right frontal lobe, the left occipital lobe, and the left caudate. Details can be found in Table 4 and Figure 1. Boxplots of each (marginally) significant cluster in Table 4 can be found in the supplementary material. The categorical effects remained robustly significant in the alternative dimensional and hybrid models, as well as in the sensitivity analyses. More details can be found in the supplementary materials.

**Table 4:** Clusters showing significant functional connectivity differences between ADHD and controls.

	Comparisons	Cluster	Voxels	Coordinate (peak voxel)	*t*-statistics (Peak)	Regions (size of overlap >10 voxels)
Default Mode Network	ADHD-I < Control	1	777	34-28 6	4.08	Right insular cortex, right Heschl’s gyrus, right planum temporale, right thalamus, right putamen, right pallidum, right hippocampus;
		2	411	−8 30 0	4.78	Left frontal pole, left superior frontal gyrus, left paracingulate gyrus; anterior cingulate gyrus;
		3	37	−28-2 12	4.36	Left putamen;
		4	34	28-14 18	3.64	Right putamen;
	ADHD-C < Control	1	926	14-4 42	4.39	Right precentral gyrus, right postcentral gyrus, right supplementary motor cortex, anterior and posterior cingulate gyrus;
		2	699	−28-4 10	5.04	Left insular cortex, left central opercular cortex, left Heschl’s gyrus, left putamen;
		3	665	46-24 44	4.87	Right precentral gyrus, right postcentral gyrus, right supramarginal gyrus,
		4	13	18-30 70	3.44	Right precentral gyrus, right postcentral gyrus;
Dorsal Attention Network	ADHD-I < Control	1	577	42 48 14	4.05	Right frontal pole, right middle frontal gyrus;
		2	75	52-34 26	4.21	Right supramarginal gyrus, right parietal operculum, right planum temporale;
	ADHD-I < ADHD-C^*^	1	122	44-52 4	4.19	Right middle temporal gyrus, right inferior temporal gyrus, right supramarginal gyrus, right angular gyrus;
		2	12	44-42-2	3.72	Right middle temporal gyrus;
Salience Network	ADHD-RI < Control^*^	1	81	−20-90-4	4.06	Left occipital pole;
	ADHD-RI < ADHD-I^*^	1	97	−8 50 20	4.17	Left superior frontal gyrus, left paracingulate gyrus;
Executive Control Network	ADHD-C < Control^*^	1	833	12-18 -6	4.11	Right thalamus, right pallidum, right accumbens, brain-stem;
		2	768	48 4 38	3.88	Right superior frontal gyrus, right middle frontal gyrus, right precentral gyrus, right postcentral gyrus, right central opercular cortex,
		3	100	−24-68 26	4.62	Left lateral occipital cortex, left cuneal cortex, precuneus cortex;
		4	82	−20-14 20	3.32	Left caudate, left putamen;
		5	56	28-4-10	3.43	Right amygdala, right putamen;
		6	14	−20 2-14	3.83	Left putamen;
		7	12	68-20 28	4.24	Right precentral gyrus, right supramarginal gyrus;

ADHD-RI, ADHD restrictive inattentive presentation, with six or more inattentive symptoms and fewer than three hyperactive/impulsive symptoms; ADHD-I, ADHD inattentive presentation, with six or more inattentive symptoms and three to five hyperactive/impulsive symptoms; ADHD-C, ADHD combined presentation, with six or more inattentive symptoms and six or more hyperactive/impulsive symptoms; HC, healthy controls. The degrees of freedom in the general linear models were 291.

* Indicated that those are clusters marginally significant, that is with a *p*-value between 0.05 to 1 (0.05 < *p* < 0.1).

## Discussion

Our study explored ADHD-RI presentation in terms of clinical features, cognitive function, and brain functional connectivity. The results showed that the ADHD-RI presentation has the fewest clinical problems and the least impaired response inhibition, albeit the most impaired sustained attention, compared to the ADHD-C and the ADHD-I presentations. The ADHD-C and the ADHD-I groups presented abnormal functional connectivity within the DMN compared to the healthy controls. Additionally, the ADHD-I group displayed abnormal functional connectivity within the dorsal attention networks compared to the control group. As for the ADHD-RI group, we failed to detect any significant alterations in functional connectivity compared to the healthy controls and the other two ADHD presentations. However, to a more lenient significant level, the ADHD-RI showed marginally significantly reduced functional connectivity within the salience network compared to the ADHD-I presentation and the control group. Taking all these results together, we inferred that the ADHD-RI presentation might have less impaired functional connectivity in the DMN and more profound functional connectivity alterations within the salience network compared to the ADHD-I and the ADHD-C presentations.

Previous studies have shown that patients with ADHD-PI (including ADHD-I and ADHD-RI) had fewer clinical problems than those with ADHD-C presentation (10). Those individuals with ADHD-C tend to have more delinquent and aggressive behaviors (10), which was replicated in the current study. In addition, we also showed that those individuals with ADHD-RI presented even fewer clinical problems. This is in line with previous findings showing a more pronounced relationship between aggressive behaviors and hyperactive/impulsive symptoms than inattentive symptoms (27). Since the ADHD-RI presentation has the fewest H/I symptoms, it is reasonable for the patients with ADHD-RI to have the fewest delinquent and aggressive behaviors among the three ADHD subtypes. The ADHD-C group also scored higher in the attention problems factor from CBCL compared to the ADHD-RI group. Our previous publication noticed the elevated score in the attention problems factor in the ADHD-C group, compared to the ADHD-PI group (10). The fact that the attention problems factor includes core symptoms of inattention and some other content, e.g., fine motor skill and dreaminess, might explain the elevated score we detected in the ADHD-C group. Some of the previous studies also reported more anxiety in the ADHD-PI presentation in contrast to the ADHD-C presentation (10, 28, 29). The current study, however, did not detect any significant between-group differences in the anxiety factor among the three ADHD groups. One of the possible reasons is that the anxious/depressed factor of CBCL mainly includes items about specific emotions (17), e.g., feeling lonely, fear of certain animals or places, feeling worthless or inferior, and so on, which is different from the anxiety factor in the Conners’ parent rating scale (21), which includes items more like an “anxious personality traits,” e.g., being shy, worries more, fears more, and is easily pushed around.

In terms of cognitive function, comparisons between the ADHD groups and the control group indicated weaker response inhibition in the ADHD-C group and weaker sustained attention in the ADHD-RI. A longitudinal study has demonstrated that the inattentive symptoms might in fact be exacerbated by engagement in aggression behaviors, which is closely related to hyperactive/impulsive symptoms (27). A genetic study also demonstrated a stronger genetic correlation between sustained attention and inattention symptoms (r = 0.64) than hyperactivity/impulsivity (*r* = 0.31) (5). Therefore, the inattentive symptoms in the ADHD-C group, or even the ADHD-I group, might be “exacerbated” by the H/I symptoms. Therefore, the original dysfunction in sustained attention is not as impaired as we expected. In fact, our results are in line with several previous studies, which demonstrated more impaired early-stage attention control, and slower processing speed in subjects with ADHD-RI compared to those with ADHD-C or ADHD-I (13, 14). Unlike the sustained attention and response inhibition domains, we did not identify any significant differences among the three ADHD groups in the spatial working memory domain, which is inconsistent with the results from a previous study. It was shown that children with ADHD-C had weak short-term visual–spatial memory and equally pronounced motivational deficits, in contrast to children with ADHD-I (30). Note that the ADHD-RI group in that study was defined to have six or more inattentive symptoms and three or fewer hyperactive/impulsive symptoms. Apart from that, the test we used in the current study, although it has been widely used (31–33), seems to be less sensitive to ADHD-related impairment in our sample, as we had shown in our previous publications (20).

The default mode network, the salience network, the executive control network, and the dorsal and ventral attention networks have been suggested to be involved in the pathophysiology of ADHD (34–37). The current study also revealed reduced functional connectivity within DMN in both the ADHD-I and the ADHD-C groups compared to the control group. Nevertheless, the difference between the ADHD-RI and the control group in terms of functional connectivity within DMN is not significant. In a recent work including 34 children and adolescence with ADHD, the ADHD-C subtype was revealed to have more profoundly reduced functional connectivity in the DMN, the dorsal attention network, and the ventral attention network (38). That study failed to detect any significant difference in functional coupling in the DMN between the ADHD-I and the control groups. The correlation between the hyperactive/impulsive and functional connectivity in the DMN was reported before (38). In the current study, the ADHD-C group had the highest hyperactive/impulsive or inattentive symptom severity, and the ADHD-RI group had the lowest hyperactive/impulsive symptom severity. That might explain why the ADHD-RI group was not significantly different from the control group. The regions showing altered functional connectivity in the ADHD-I or the ADHD-C group, in contrast to the control group, were different. One of the possible explanations for this phenomenon is that the neurobiological underpinnings for ADHD are both dimensional (correlating with symptom counts) and categorical (diagnosis sensitive) or even hybrid (interaction of categorical and dimensional effects). Future studies in clinical samples or, ideally, the general population is warranted to further test this hypothesis.

Besides the DMN, the ADHD-I group also presented altered brain functional connectivity in the dorsal attention network (DAN). Previous study comparing individuals with ADHD-PI and those with ADHD-C to healthy controls has identified reduced functional connectivity in the left inferior occipital gyrus and right superior occipital gyrus of the DAN in the ADHD-C group and the right superior parietal gyrus of the DAN in the ADHD-PI group (39). We did not detect any alterations in functional connectivity in the ADHD-C or the ADHD-RI group compared to the healthy controls. As mentioned above, the inattentive symptoms might be “exacerbated” by the hyperactive/impulsive symptoms. Therefore, ADHD-C might not have as much inattention-related brain functional abnormality as we expected, according to their inattentive clinical symptoms. While the hyperactivity/impulsivity symptoms cause extra impairments in the DMN, they might also attenuate the impairments in the DAN. Supporting this theory, our group has identified different patterns of structural and microstructural alterations in the ADHD-PI and the ADHD-C subtypes compared to healthy controls, with the ADHD-PI but not the ADHD-C presentation showing altered white matter integrity (10). The salience network was thought to mediate attention to salient internal/external stimuli, guiding behavior (40). The functional connectivity between the dorsal anterior cingulate cortex and anterior insular was found to be related to self-reported ADHD symptoms in individuals with ADH (40). Consistent with this, the current study detected marginally significantly reduced functional connectivity of the ADHD-RI group, compared to the ADHD-I and the control groups.

### Limitations

The current study explored the clinical, neuropsychological, and brain-functional characteristics of the ADHD-RI presentation in comparison to healthy controls, the ADHD-I, and the ADHD-C presentations. In our sample, the ADHD groups have more male subjects than the control group, which might restrain the generalization of the results of the current study. The ADHD and control groups differed significantly in terms of age, and our exploration analyses of the interacting effects of diagnosis and age have revealed that the between-group differences might be affected by age. Future study with more age-matched sample or longitudinal design is warranted.

## Conclusion

In summary, the current study demonstrated fewer clinical problems, weaker sustained attention, better response inhibition, fewer alterations in the functional connectivity in the DMN, and potentially more impaired brain function in the salience network. The current study contributes to the ongoing debates on ADHD-RI presentations. The results supported the notion of ADHD-RI being an independent presentation. We also noticed the complicated interactive effects, rather than simply additive or aggregative effects, of the two ADHD symptom domains regarding cognition and brain functional connectivity. It is not a severity continuum. Aggregation, compensation, and additive and interactive effects of these two symptom domains may all play a role in causing functional brain alterations in patients with ADHD. Future studies using brain structural and functional information to subtype ADHD patients might provide more insights. In addition, future studies exploring the neurological underpinnings of ADHD symptoms in the general population and clinical samples, embracing both the categorical, dimensional, and hybrid models, are also warranted.

## Data availability statement

The original contributions presented in the study are included in the article/Supplementary material, further inquiries can be directed to the corresponding authors.

## Ethics statement

The studies involving human participants were reviewed and approved by the Ethics committee of Shenzhen Children’s Hospital. Written informed consent to participate in this study was provided by the participants’ legal guardian/next of kin.

## Author contributions

Z-MW and PW contributed significantly to the data collection, data analysis, and manuscript preparation. JL and X-LC helped to conduct the study. LL and LS helped with the data analysis, as well as the writing and reviewing of the manuscript. LY, Q-JC, B-RY, and Y-FW contributed to the design of the study, the data analysis, and the manuscript preparation. All authors contributed to the article and approved the submitted version.

## Funding

This work was supported by the National Natural Science Foundation of China (82101613 and 82071537) and the SANMING Project of Medicine in Shenzhen “The ADHD research group from Peking University Sixth Hospital” (SZSM201612036).

This work was also supported by the Guangdong High-level Hospital Construction Fund.

## Conflict of interest

The authors declare that the research was conducted in the absence of any commercial or financial relationships that could be construed as a potential conflict of interest.

## Publisher’s note

All claims expressed in this article are solely those of the authors and do not necessarily represent those of their affiliated organizations, or those of the publisher, the editors and the reviewers. Any product that may be evaluated in this article, or claim that may be made by its manufacturer, is not guaranteed or endorsed by the publisher.

## Footnotes

^1^ https://www.cambridgecognition.com/cantab/cognitive-tests

^2^ http://fsl.fmrib.ox.ac.uk/fsl

^3^ https://www.python.org/

^4^ https://cma.mgh.harvard.edu/
