# Supplementary material for: The clinical, neuropsychological, and brain functional characteristics of the ADHD restrictive inattentive presentation
Source: Front Psychiatry. 2023 Mar 1;14:1099882. doi: 10.3389/fpsyt.2023.1099882 (PMC10014598; doi:10.3389/fpsyt.2023.1099882)
Supplement: Supplementary file 1 [file Data_Sheet_1.docx]

Supplementary Material

## Methods

**Statistical Analyses**: 1) To build the alternative dimensional models, we extracted the mean functional connectivity (FC) values of those clusters showing significant or marginally significant group-wise differences. Linear regression models were performed with symptom severity (inattention or hyperactivity/impulsivity separately) as the independent variable, and sex and age were added as covariates. Bonferroni's correction was applied by dividing 0.05 by the number of significant clusters within the same brain functional networks. Therefore, the significance level was set to 0.05/8 = 0.00625 for the DMN, 0.05/4=0.0125 for the DAN, 0.05/2=0/025 for the Salience Network, and 0.05/7=0.00714 for the ECN. Since the potential interaction between diagnosis and symptom severity, the dimensional models were tested within the ADHD group (with all ADHD subjects) and each ADHD subgroup. 2) For those clusters showing significant (Bonferroni corrected) dimensional effects, hybrid models were built with both diagnosis (ADHD presentations) and symptom severity (inattention or hyperactivity/impulsivity separately) as the independent variables. The interaction variables were added only when the dimensional effects were different among the three ADHD subgroups. Sex and age were also added as covariates. 3) To further eliminate the potential head motion artifact, the root mean squared of the relative displacement time series (RMS) was added as an additional covariate.4) To further test the confounding effects of age, the age^2 (age square) was added as an additional covariate to the original models. 5) To further illustrate the potential interaction between diagnosis and age, which would lead to confounding effects, the interaction effects were tested within each brain network in a voxel-wise manner, focusing on those comparisons showing significant between-group differences in the original analysis. For those brain networks showing significant interaction effects, the mean FC values of those clusters showing significant group-wise differences were extracted, and linear models with diagnosis, age, and the interaction between age and diagnosis as independent variables were tested. 6) The brain-behavior and brain-cognition relationships were also estimated within each ADHD group using linear models, with age and sex added as covariates. To correct for multiple comparisons, the significance level was set to 0.05/(number of clusters within each brain network × number of measures in each cognitive domain). For each CBCL factor, the significant level was set to 0.000781 for clusters within the DMN and the ECN, 0.00156 for clusters within DAN, and 0,00313 for clusters within the salience network. For each RVP and RTI measure, the significant level was set to 0.00156 for clusters within the DMN and the ECN, 0.00313 for clusters within DAN, and 0.00625 for clusters within the salience network. For each SST measure, the significant level was set to 0.00208 for clusters within the DMN and the ECN, 0.00416 for clusters within DAN, and 0.00833 for clusters within the salience network. For each SWM measure, the significant level was set to 0.00313 for clusters within the DMN and the ECN, 0.00625 for clusters within DAN, and 0.0125 for clusters within the salience network.

**ADHD outpatient clinics**

**Clinical interviews**

**Semi-structured interviews**

**Behavioral checklists**

**And cognitive tests**

**Inclusion criteria**:1) aged 6-15 years;

2) educated in public/ordinary private schools;

3) a diagnosis of ADHD.

**Exclusion criteria**: 1) a history of head injury with loss of consciousness;

2) any severe physical disease or neurological abnormalities;

3) any kind of drug or substance misuse;

4) a full-scale IQ measured by Wechsler Intelligence Scale for Chinese Children-IV (WISC-IV) below 70;

5) Long-term use of any prescribed medications for ADHD or other medical conditions.

**MRI scans**

**Inclusion criteria**:1) right-hand dominant; 2) informed consent obtained;

**Exclusion criteria**:1) any visible abnormalities (e.g., arachnoid cyst) on the MRI images (17 individuals); 2) excessive head motion (5 individuals were excluded during the scan, and 4 more individuals were excluded during the data processing step); 3) a past or current history of claustrophobia (4 individuals);

sFigure 1: The flow chart of inclusion and exclusion of the ADHD subjects

**Students in elementary school**

**Clinical interviews**

**Semi-structured interviews**

**Behavioral checklists**

**And cognitive tests**

**Inclusion criteria**:1) aged 6-15 years;

2) educated in public/ordinary private schools;

**Exclusion criteria**: 1) a history of head injury with loss of consciousness;

2) any severe physical disease or neurological abnormalities;

3) any kind of drug or substance misuse;

4) a full-scale IQ measured by Wechsler Intelligence Scale for Chinese Children-IV (WISC-IV) below 70;

5) Long-term use of any prescribed medications for ADHD or other medical conditions.

6) a past or current history of any psychiatric disorder;

**MRI scans**

**Inclusion criteria**:1) right-hand dominant; 2) informed consent obtained;

**Exclusion criteria**:1) any visible abnormalities (e.g., arachnoid cyst) on the MRI images (8 individuals); 2) excessive head motion (no one was excluded at this step); 3) a past or current history of claustrophobia(no one was excluded at this step);

sFigure 2: The flow chart of inclusion and exclusion of the control subjects


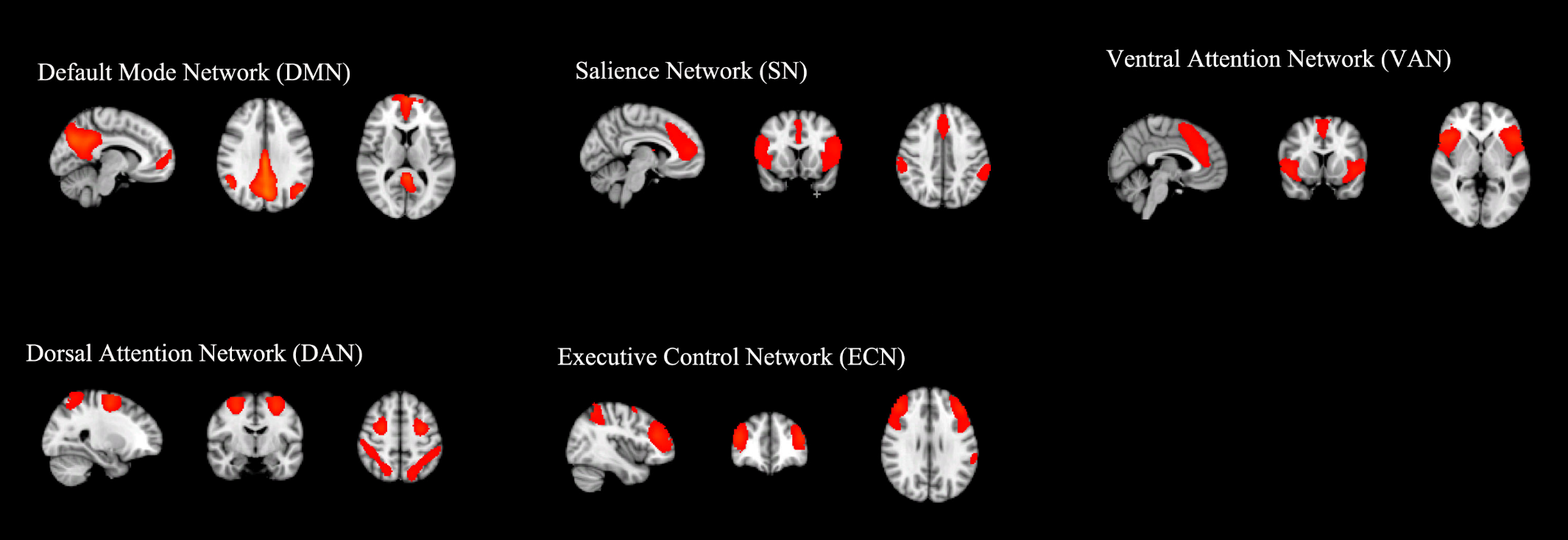


sFigure 3: Spatial maps of the five brain networks (red), including the default mode network (DMN), the dorsal attention network (DAN), the ventral attention network (VAN), the executive control network (ECN), and the salience network (SN), displayed on a MNI152_T1_2mm brain (grey)..

## Results

**Dimensional and Hybrid models:** 1) After correcting for multiple comparisons, the mean FC value of the ADHD-I <ADHD-C comparison cluster 1 in the DAN was negatively associated with the inattention score (beta = -0.030, *p* = 0.0121) within the ADHD-C group. Results of the hybrid models with diagnosis, inattention symptom severity, and their interactions as independent variables revealed significant categorical, dimensional, and interacting effects (beta = -1.20, *p* = 0.00235 for the diagnosis of ADHD-I, beta = -0.029, *p* = 0.00860 for the inattention score, and beta = 0.044, p = 0.00601 for their interaction). 2) The mean FC value of ADHD-I < ADHD-C comparison cluster 2 in the DAN (beta = 0.0046, *p* = 0.0042) and the mean FC value of ADHD-C < Control Cluster 1 in the ECN (beta = -0.0027, *p* = 0.00209) were associated with the hyperactivity/impulsivity score within the whole ADHD group. The hybrid model of the mean FC value of ADHD-C < Control Cluster 1 in the ECN demonstrated significant dimensional effects (beta = -0.0051, *p* = 0.010) and non-significant categorical effects (all *p* > 0.05). However, the association between hyperactivity/impulsivity score and the mean FC value of ADHD-I < ADHD-C comparison cluster 2 in the DAN was no longer significant (*p* = 0.477). Details are summarized in sTable 1.

sTable 1: Dimensional effects of clusters showing significant group-wise differences (beta(p-value))

| Brain Networks |  | Cluster | ADHD-RI | | ADHD-PI | | ADHD-C | | | Whole ADHD group | |
| --- | --- | --- | --- | --- | --- | --- | --- | --- | --- | --- | --- |
|  |  |  | ATT | H/I | ATT | H/I | ATT | H/I | ATT | | H/I |
| DMN | ADHD-I < Control | 1 | 0.00052(0.964) | -0.00042(0.960) | 0.0078(0.270) | -0.0022(0.719) | -0.0040(0.423) | -0.0012(0.818) | 0.00076(0.850) | | -0.0000072 (0.996) |
|  |  | 2 | 0.018(0.476) | 0.028(0.0885) | 0.0076(0.610) | -0.0085(0.504) | 0.019(0.181) | 0.0040(0.777) | 0.016(0.0958) | | **0.0072(0.0440)** |
|  |  | 3 | 0.0025(0.832) | 0.0065(0.447) | 0.0097(0.262) | -0.0091(0.217) | -0.0071(0.402) | -0.0051(0.554) | -0.0017(0.748) | | **-0.0043(0.0355)** |
|  |  | 4 | **0.022(0.0132)** | 0.0022(0.739) | 0.0083(0.195) | -0.0084(0.122) | -0.0071(0.263) | -0.0075(0.242) | 0.0047(0.228) | | -0.0018(0.232) |
|  | ADHD-C < Control | 1 | -0.0065(0.731) | 0.0071(0.608) | -0.0010(0.307) | **-0.020(0.0177)** | 0.0090(0.440) | -0.010(0.382) | -0.0031(0.664) | | **-0.0072(0.0088)** |
|  |  | 2 | -0.00052(0.966) | 0.0055(0.532) | -0.0011(0.889) | -0.0061(0.374) | -0.0072(0.392) | -0.0097(0.258) | -0.0064(0.218) | | **-0.0053(0.00892)** |
|  |  | 3 | 0.0021(0.932) | 0.016(0.380) | -0.0074(0.687) | -0.030(0.0521) | 0.00019(0.993) | -0.014(0.532) | -0.0069(0.583) | | **-0.012(0.0187)** |
|  |  | 4 | 0.020(0.557) | 0.0051(0.822) | -0.044(0.1427) | **-0.053(0.0386)** | -0.0058(0.847) | -0.018(0.559) | -0.020(0.262) | | **-0.015(0.0337)** |
| DAN | ADHD-I < Control | 1 | -0.020(0.260) | 0.0034(0.798) | 0.013(0.435) | -0.00058(0.967) | 0.010(0.364) | -0.0048(0.669) | 0.0077(0.358) | | 0.00055(0.868) |
|  |  | 2 | 0.013(0.642) | -0.012(0.530) | 0.0078(0.723) | -0.0073(0.695) | -0.015(0.340) | 0.0020(0.902) | 0.00037(0.975) | | -0.00089(0.845) |
|  | ADHD-I < ADHD-C | 1 | -0.00014(0.993) | -0.0040(0.740) | 0.012(0.278) | 0.010(0.270) | **-0.030(0.0121)*** | -0.0016(0.893) | -0.0014(0.855) | | **0.0069(0.0172)** |
|  |  | 2 | 0.0041(0.701) | -0.00055(0.941) | 0.010(0.133) | 0.0091(0.124) | 0.0041(0.508) | -0.0030(0.636) | **0.0087(0.0382)** | | **0.0046(0.00420)*** |
| Salience | ADHD-RI < Control | 1 | -0.010(0.843) | -0.019(0.613) | 0.015(0.284) | -0.012(0.347) | 0.020(0.192) | -0.0087(0.568) | 0.024(0.0811) | | 0.0067(0.222) |
|  | ADHD-RI < ADHD-I | 1 | **-0.030(0.027)** | -0.0046(0.657) | -0.0027(0.769) | **-0.017(0.0287)** | 0.0058(0.573) | 0.0053(0.610) | -0.00030(0.961) | | 0.0036(0.146) |
| ECN | ADHD-C < Control | 1 | 0.0038(0.413) | -0.0042(0.214) | -0.00039(0.908) | -0.0044(0.130) | -0.0035(0.378) | -0.0062(0.122) | -0.0015(0.516) | | **-0.0027(0.00209)*** |
|  |  | 2 | -0.016(0.233) | -0.018(0.0672) | -0.016(0.157) | 0.00059(0.952) | 0.013(0.196) | **-0.021(0.0396)** | -0.0024(0.713) | | **-0.0057(0.0233)** |
|  |  | 3 | -0.016(0.222) | 0.000028(0.998) | -0.014(0.133) | -0.0012(0.873) | 0.014(0.157) | -0.0040(0.684) | -0.0031(0.597) | | -0.0036(0.113) |
|  |  | 4 | -0.0018(0.684) | -0.0028(0.383) | -0.0046(0.206) | -0.0020(0.516) | -0.0049(0.172) | -0.0022(0.541) | -0.0036(0.0917) | | -0.00035(0.682) |
|  |  | 5 | -0.0024(0.770) | -0.011(0.0692) | **-0.013(0.0475)** | -0.0042(0.457) | 0.0021(0.739) | -0.0060(0.351) | -0.0055(0.157) | | **-0.0036(0.0174)** |
|  |  | 6 | 0.0012(0.927) | **-0.018(0.0491)** | -0.013(0.198) | -0.0031(0.718) | 0.0024(0.774) | -0.0088(0.297) | -0.0050(0.362) | | -0.0037(0.0852) |
|  |  | 7 | -0.010(0.628) | 0.013(0.392) | -0.0046(0.744) | -0.012(0.329) | 0.014(0.387) | -0.017(0.279) | 0.0020(0.831) | | **-0.0085(0.0176)** |

Note: All nominally significant results (p < 0.05) were presented in bold. * indicated the results remained significant after Bonferroni correction;

Abbreviations: ADHD-RI: ADHD restrictive inattentive presentation, with six or more inattentive symptoms and fewer than three hyperactive/impulsive symptoms; ADHD-I: ADHD inattentive presentation, with six or more inattentive symptoms and three to five hyperactive/impulsive symptoms; ADHD-C: ADHD combined presentation, with six or more inattentive symptoms and fewer than three hyperactive/impulsive symptoms; HC: healthy controls; DMN: default mode network; DAN: dorsal attention network; ECN: executive control network; ATT: inattention symptom severity; H/I: hyperactivity/impulsivity symptom severity.

**Sensitivity Analyses:** By adding the head motion parameter or age^2 as additional covariates, the results did not change significantly. Results were summarized in sTable 2 (head motion) and sTable 3 (age^2).

sTable 2 Clusters showing significant functional connectivity differences between ADHD and controls (with head motion parameter as an additional covariate)

|  | Comparisons | Cluster | Voxels | Coordinate (peak voxel) | Regions (size of overlap > 10 voxels) |
| --- | --- | --- | --- | --- | --- |
| Default Mode Network | ADHD-I < Control | 1 | 999 | 34 -28 4 | Right insular cortex, right Heschl’s gyrus, right planum temporale, right parietal operculum cortex, right thalamus, right putamen, right pallidum, right hippocampus; |
|  |  | 2 | 290 | -8 30 0 | Left frontal pole, left paracingulate gyrus, anterior cingulate gyrus; |
|  |  | 3 | 67 | -28 -2 12 | Left insular cortex, left putamen; |
|  |  | 4 | 33 | -34 -22 6 | Left insular cortex, left Heschel’s gyrus; |
|  |  | 5 | 25 | 2 46 16 | Right central opercular cortex; |
|  | ADHD-C < Control | 1 | 498 | -24 4 12 | Left insular cortex, left central opercular cortex, left parietal operculum cortex, left Heschl’s gyrus, left putamen; |
|  |  | 2 | 362 | 46 -24 44 | Right precentral gyrus, right postcentral gyrus, right supramarginal gyrus; |
|  |  | 3 | 287 | -8 -14 54 | Left precentral gyrus, left postcentral gyrus, left supplementary motor cortex, anterior and posterior cingulate cortex; |
|  |  | 4 | 228 | 14 -4 44 | Right precentral gyrus, right supplementary motor cortex, anterior and posterior cingulate cortex; |
| Dorsal Attention Network | ADHD-I < Control | 1 | 522 | 42 46 8 | Right frontal pole, right middle frontal gyrus; |
|  |  | 2 | 75 | 52 -34 26 | Right supramarginal gyrus, right parietal operculum cortex, right planum temporale; |
|  | ADHD-I < ADHD-C^*^ | 1 | 8 | 44 -52 4 | Right middle temporal gyrus; |
| Salience Network | ADHD-RI < Control^*^ | 1 | 81 | -20 -90 -4 | Left inferior occipital cortex, left occipital pole, left occipital fusiform gyrus, left lingual gyrus, left intracalcarine cortex; |
|  | ADHD-RI < ADHD-I^*^ | 1 | 86 | -4 54 26 | Left frontal pole, left superior frontal gyrus, left paracingulate gyrus; |
| Executive Control Network | ADHD-C < Control^*^ | 1 | 1210 | 12 -20 -6 | Left thalamus, brain-stem, right thalamus, right putamen, right pallidum, right accumbens, |
|  |  | 2 | 649 | 46 2 36 | Right superior frontal gyrus, right middle frontal gyrus, right inferior frontal gyrus, right precentral gyrus, right postcentral gyrus, right central opercular cortex; |
|  |  | 3 | 160 | -22 -14 20 | Left thalamus, left caudate, left putamen; |
|  |  | 4 | 140 | -24 -66 26 | Left lateral occipital cortex, left precuneous cortex, left cuneal cortex, left supracalcarine cortex; |
|  |  | 5 | 44 | -10 -10 2 | Left thalamus; |
|  |  | 6 | 36 | 18 -12 20 | Right thalamus, right caudate; |
|  |  | 7 | 14 | -20 2 -14 | Left putamen, left frontal orbital cortex; |

Note: * indicated that those are clusters marginally significant, that is with a *p*-value between 0.05 to 1 (0.05 < *p* < 0.1);

Abbreviations: ADHD-RI: ADHD restrictive inattentive presentation, with six or more inattentive symptoms and fewer than three hyperactive/impulsive symptoms; ADHD-I: ADHD inattentive presentation, with six or more inattentive symptoms and three to five hyperactive/impulsive symptoms; ADHD-C: ADHD combined presentation, with six or more inattentive symptoms and fewer than three hyperactive/impulsive symptoms;

Table 3 Clusters showing significant functional connectivity differences between ADHD and controls (with age^2 as an additional covariate)

|  | Comparisons | Cluster | Voxels | Coordinate (peak voxel) | Regions (size of overlap > 10 voxels) |
| --- | --- | --- | --- | --- | --- |
| Default Mode Network | ADHD-I < Control | 1 | 138 | 34 -28 6 | Right insular cortex, right Heschl’s gyrus, right planum temporale; |
|  |  | 2 | 87 | 14 -4 -6 | Right thalamus, right pallidum, right putamen; |
|  |  | 3 | 37 | -8 34 2 | Anterior cingulate gyrus, left paracingulate gyrus; |
|  |  | 4 | 21 | 30 -2 -10 | Posterior cingulate gyrus, Right hippocampus; |
|  | ADHD-C < Control | 1 | 611 | -8 -14 54 | Left precentral gyrus, left supplementary motor cortex, anterior and posterior cingulate gyrus; |
|  |  | 2 | 565 | -28 -4 10 | Left insular cortex, left central opercular cortex, left parietal operculum cortex, left Heschl’s gyrus, left putamen; |
|  |  | 3 | 445 | 46 -24 44 | Right precentral gyrus, right postcentral gyrus, right supramarginal gyrus; |
| Dorsal Attention Network | ADHD-I < Control | 1 | 577 | 42 48 16 | Right frontal pole, right middle frontal gyrus; |
|  | ADHD-I < ADHD-C^*^ | 1 | 163 | 44 -52 4 | Right middle temporal gyrus, right inferior temporal gyrus, right supramarginal gyrus, right angular gyrus; right temporal occipital fusiform; |
| Salience Network | ADHD-RI < Control^*^ | 1 | 100 | -20 -90 -4 | Left occipital pole, left occipital fusiform gyrus, left lingual gyrus, left intracalcarine cortex; |
|  |  | 2 | 49 | 50 10 36 | Right middle frontal gyrus, right precentral gyrus, right inferior frontal gyrus; |
|  | ADHD-RI < ADHD-I^*^ | 1 | 118 | -4 54 26 | Left superior frontal gyrus, left paracingulate gyrus; left frontal pole; |
| Executive Control Network | ADHD-C < Control^*^ | 1 | 1532 | 12 -18 -6 | Right thalamus, right pallidum, right pallidum, right accumbens, left thalamus, left caudate, brain-stem; |
|  |  | 2 | 903 | 48 4 38 | Right superior frontal gyrus, right middle frontal gyrus, right precentral gyrus, right postcentral gyrus, right central opercular cortex, right inferior frontal gyrus; |
|  |  | 3 | 123 | -22 -66 26 | Left lateral occipital cortex, left cuneal cortex, precuneous cortex, left supracalcarine cortex; |
|  |  | 4 | 44 | 68 -20 26 | Right supramarginal gyrus, right postcentral gyrus; |
|  |  | 5 | 38 | -20 2 -14 | Left putamen, left amygdala, left frontal orbital cortex; |
|  |  | 6 | 21 | -10 10 2 | Left thalamus; |
|  |  | 7 | 11 | 62 -38 24 | Right caudate; |

Note: * indicated that those are clusters marginally significant, that is with a *p*-value between 0.05 to 1 (0.05 < *p* < 0.1);

Abbreviations: ADHD-RI: ADHD restrictive inattentive presentation, with six or more inattentive symptoms and fewer than three hyperactive/impulsive symptoms; ADHD-I: ADHD inattentive presentation, with six or more inattentive symptoms and three to five hyperactive/impulsive symptoms; ADHD-C: ADHD combined presentation, with six or more inattentive symptoms and fewer than three hyperactive/impulsive symptoms; HC: healthy controls;

**Interaction between Age and Diagnosis:** In the voxel-based analysis, the interaction between diagnosis and age was significant in the compassions between the ADHD-RI group and the ADHD-I and the control group in the Salience Network (p < 0.05). Further analysis revealed a significant effect of the diagnosis * age interaction for the two marginally significant clusters in the Salience Network (the ADHD-RI < ADHD-I cluster: beta = 0.22, *p* = 0.180; the ADHD-RI < Control cluster: beta = -0.12, *p* = 0.0070). The main effect of diagnosis on the mean FC value of the ADHD-RI < ADHD-PI cluster was no longer significant (beta = 0.22, *p* = 0.180). The main effect of diagnosis on the mean FC value of the ADHD-RI < Control cluster remained significant (beta = 0.86, *p* = 0.0324). The effect of diagnosis was no longer significant when the diagnosis * age interaction was added to the linear regression model. Details can be found in sFigure 4 and sFigure 5.
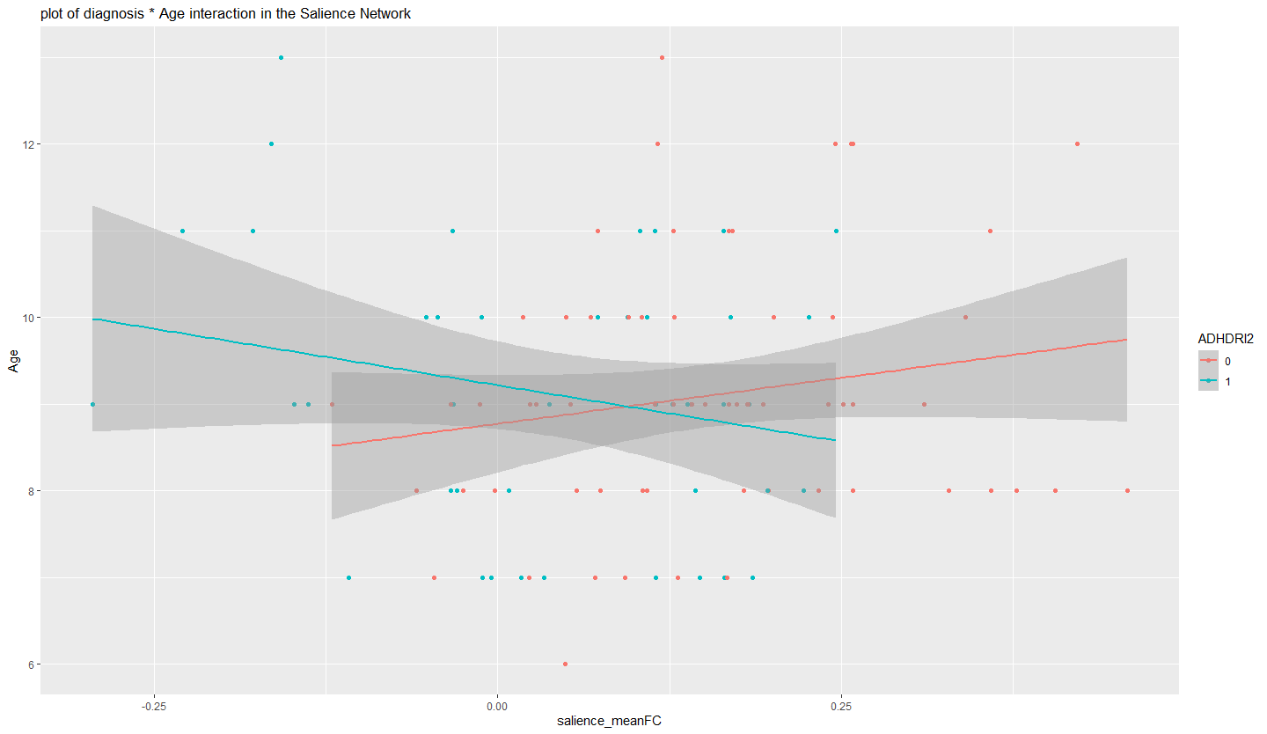


sFigure 4: Interaction between age and diagnosis (ADHD-RI vs. ADHD-I) and correlation between mean functional connectivity value of the ADHD-RI < ADHD-I cluster in the Salience Network within the ADHD-RI (turquoise line) and the ADHD-I (red line) groups. Abbreviations: ADHD-RI =ADHD restrictive inattentive presentation; ADHD-I = ADHD inattentive presentation;


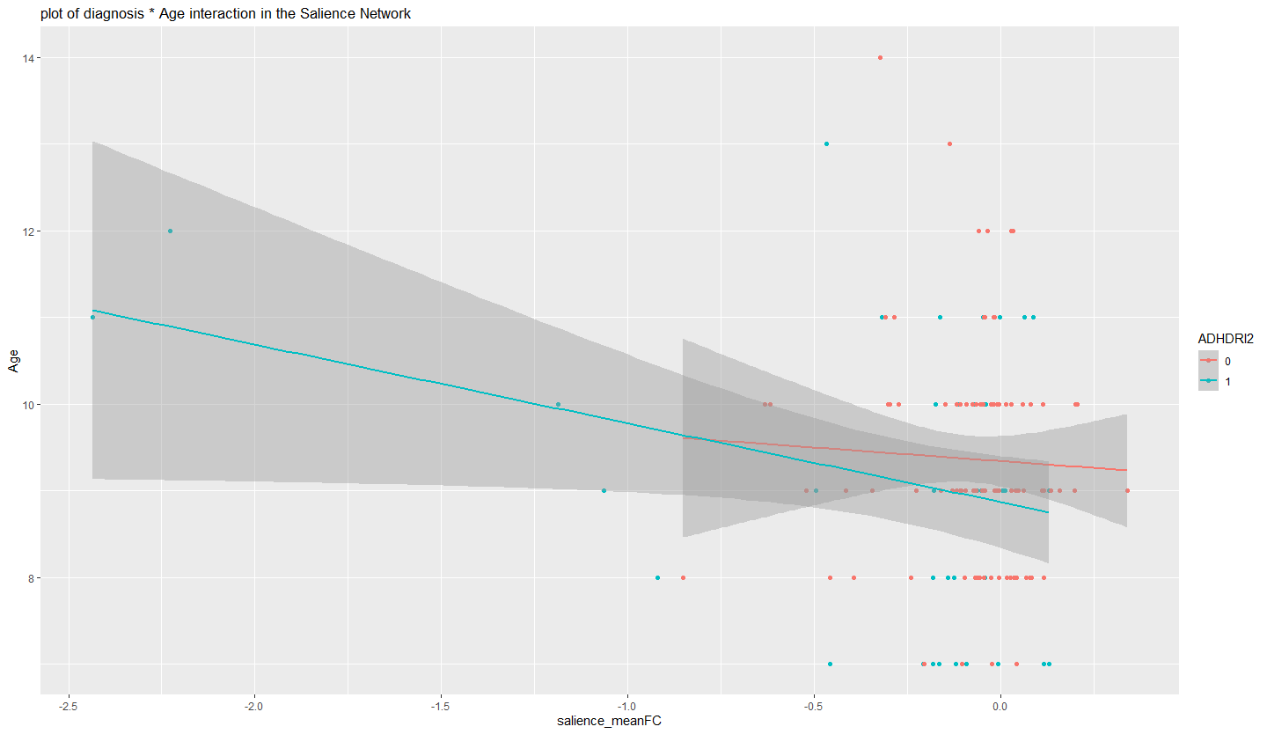


sFigure 5: Interaction between age and diagnosis (ADHD-RI vs. Control) and correlation between mean functional connectivity value of the ADHD-RI < Control cluster in the Salience Network within the ADHD-RI (turquoise line) and the Control (red line) groups. Abbreviations: ADHD-RI =ADHD restrictive inattentive presentation;

**Brain-behavior and brain-cognition relationships:** After correcting for multiple comparisons, one of the clusters within the DAN (ADHD-I < ADHD-C, Cluster 2) was significantly associated with the attention factor in CBCL (beta=-0.025, p=0.000251) within the ADHD-RI group. For the CANTAB measures, one of the clusters within the DMN showing significant between-group differences (ADHD-I < Control, Cluster 1) was significantly related to the RTIFRTSD measure within the (beta=-0.0021, p=0.00163) within the ADHD-RI group, one cluster within the ECN (ADHD-C < Control, Cluster1) was significantly related to the SSTDES within the ADHD-C group (beta=0.0023, p=0.00127) within the ADHD-C group, and another cluster within the ECN (ADHD-C < Control, Cluster5) was significantly associated with the SSTDEG measure (beta=0.0012, p=0.00103) within the ADHD-I group.


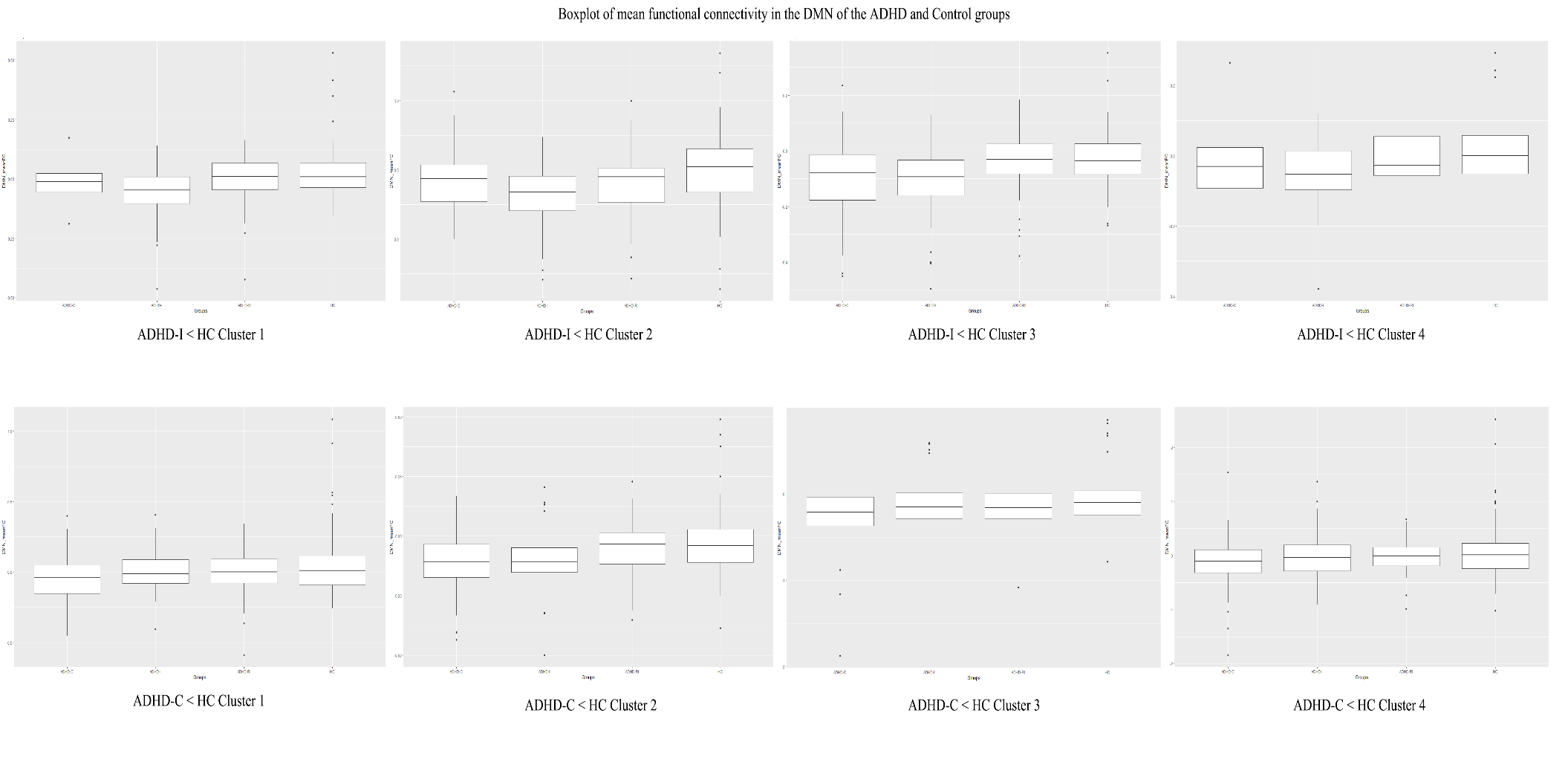


sFigure 6: Boxplots of each (marginally) significant cluster in the default mode network. Abbreviations: DMN=default mode network; ADHD-RI= ADHD restrictive inattentive presentation; ADHD-I=ADHD inattentive presentation; ADHD-C= ADHD combined presentation, with six or more inattentive symptoms and fewer than three hyperactive/impulsive symptoms; HC=healthy controls.


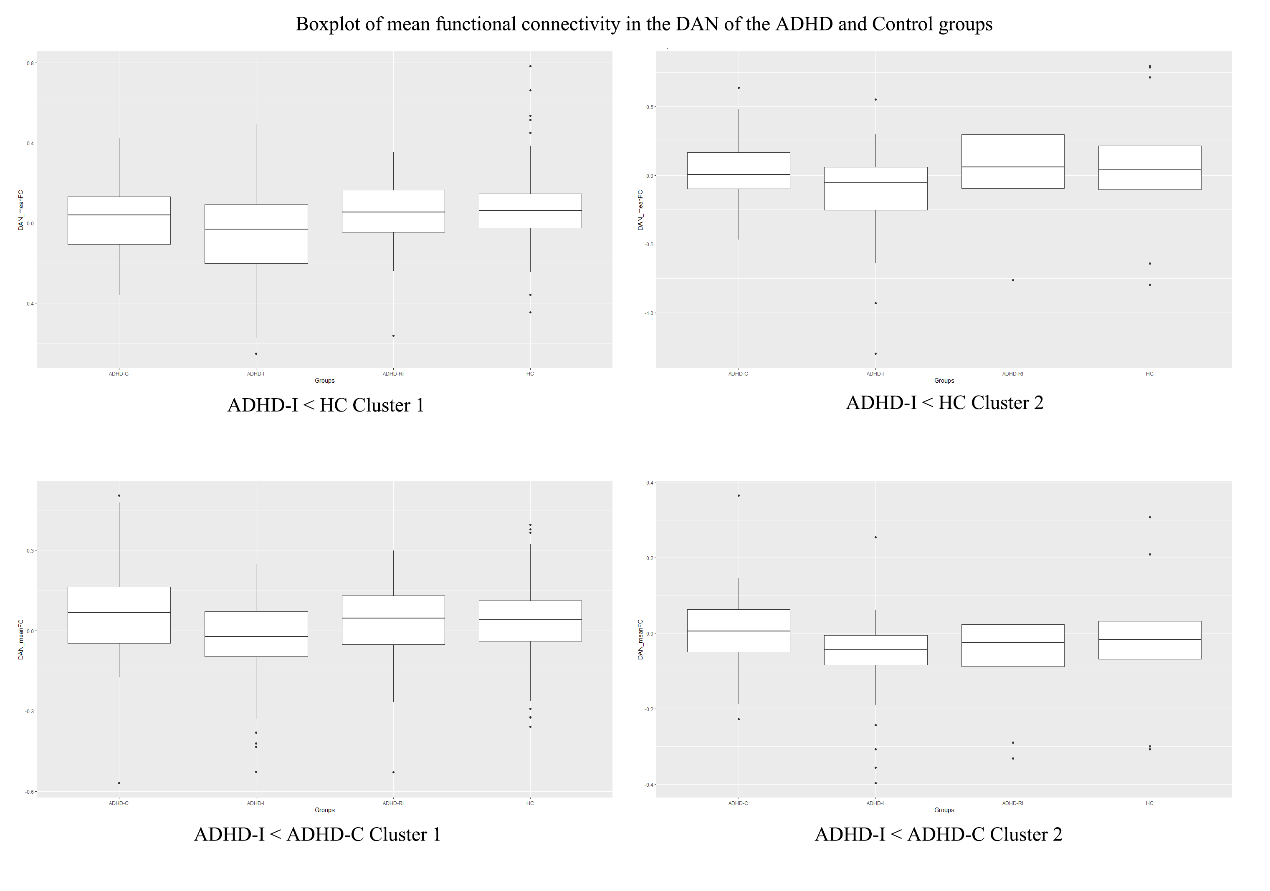


sFigure 7: Boxplots of each (marginally) significant cluster in the default mode network. Abbreviations: DAN=dorsal attention network; ADHD-RI= ADHD restrictive inattentive presentation; ADHD-I=ADHD inattentive presentation; ADHD-C= ADHD combined presentation, with six or more inattentive symptoms and fewer than three hyperactive/impulsive symptoms; HC=healthy controls.


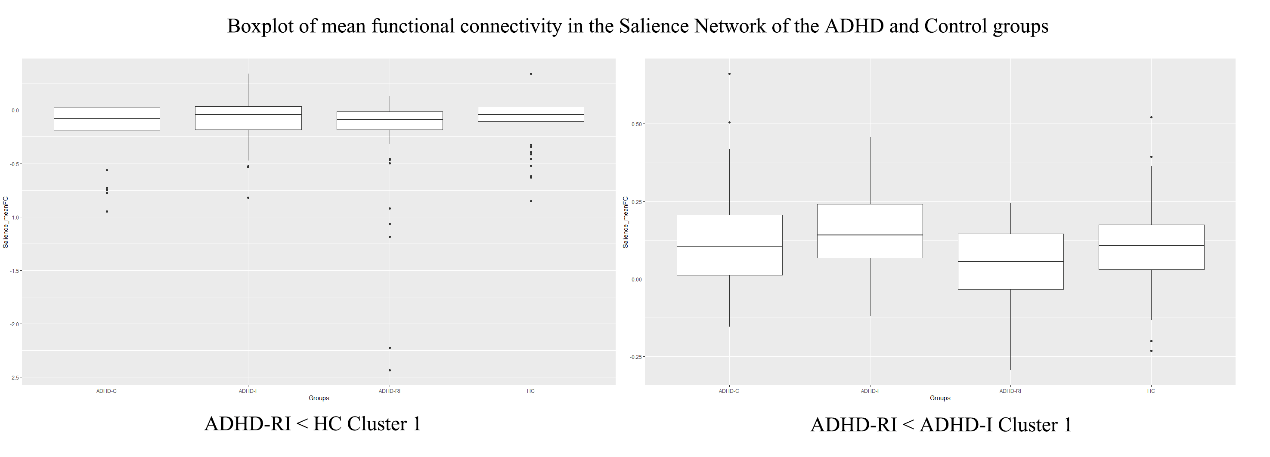


sFigure 8: Boxplots of each (marginally) significant cluster in the default mode network. Abbreviations: ADHD-RI= ADHD restrictive inattentive presentation; ADHD-I=ADHD inattentive presentation; ADHD-C= ADHD combined presentation, with six or more inattentive symptoms and fewer than three hyperactive/impulsive symptoms; HC=healthy controls.


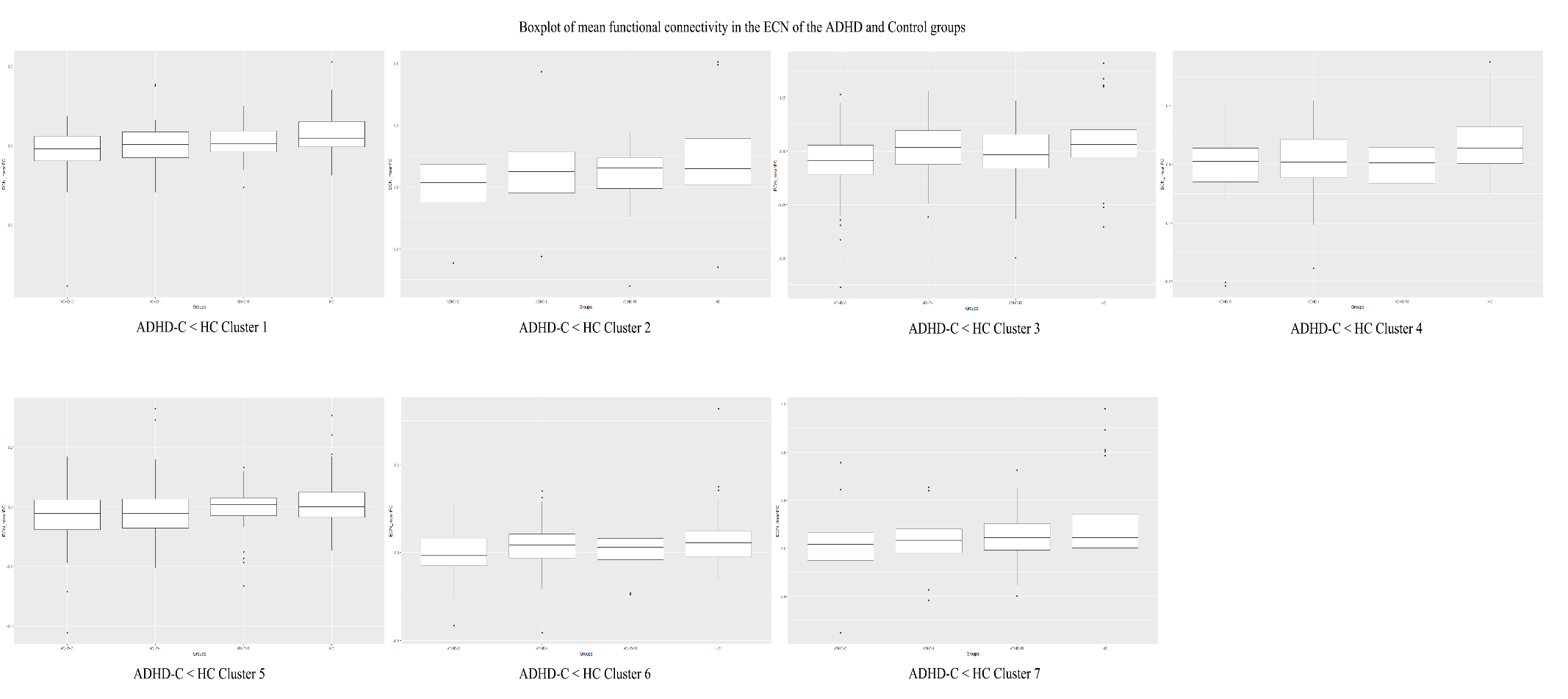


sFigure 9: Boxplots of each (marginally) significant cluster in the default mode network. Abbreviations: ECN=executive control network; ADHD-RI= ADHD restrictive inattentive presentation; ADHD-I=ADHD inattentive presentation; ADHD-C= ADHD combined presentation, with six or more inattentive symptoms and fewer than three hyperactive/impulsive symptoms; HC=healthy controls.
